# Status of racial disparities between black and white women undergoing assisted reproductive technology in the US

**DOI:** 10.1186/s12958-020-00662-4

**Published:** 2020-11-19

**Authors:** D. B. Seifer, B. Simsek, E. Wantman, A. M. Kotlyar

**Affiliations:** 1 Department of Obstetrics, Gynecology and Reproductive Sciences, Yale School of Medicine, 330 Cedar St, New Haven, CT 06510 USA; 2 Department of Statistics, University of Pittsburgh, Pittsburgh, PA 15260 USA; 3 Redshift Technologies, New York, NY 10016 USA

**Keywords:** Racial disparities, Assisted reproductive technology, Cumulative live birth rate, Health care disparities, SARTCORS database, State mandated insurance, Access-to-care

## Abstract

**Background:**

Numerous studies have demonstrated substantial differences in assisted reproductive technology outcomes between black non-Hispanic and white non-Hispanic women. We sought to determine if disparities in assisted reproductive technology outcomes between cycles from black non-Hispanic and white non-Hispanic women have changed and to identify factors that may have influenced change and determine racial differences in cumulative live birth rates.

**Methods:**

This is a retrospective cohort study of the SARTCORS database outcomes for 2014–2016 compared with those previously reported in 2004–2006 and 1999/2000. Patient demographics, etiology of infertility, and cycle outcomes were compared between black non-hispanic and white non-hispanic patients. Categorical values were compared using Chi-squared testing. Continuous variables were compared using t-test. Multiple logistic regression was used to assess confounders.

**Results:**

We analyzed 122,721 autologous, fresh, non-donor embryo cycles from 2014 to 2016 of which 13,717 cycles from black and 109,004 cycles from white women. The proportion of cycles from black women increased from 6.5 to 8.4%. Cycles from black women were almost 3 times more likely to have tubal and/or uterine factor and body mass index ≥30 kg/m^2^. Multivariate logistic regression demonstrated that black women had a lower live birth rate (OR 0.71;*P* < 0.001) and a lower cumulative live birth rate for their initial cycle (OR 0.64; *P* < 0.001) independent of age, parity, body mass index, etiology of infertility, ovarian reserve, cycle cancellation, past spontaneous abortions, use of intra-cytoplasmic sperm injection or number of embryos transferred. A lower proportion of cycles in black women were represented among non-mandated states (*P* < 0.001) and cycles in black women were associated with higher clinical live birth rates in mandated states (*P* = 0.006).

**Conclusions:**

Disparities in assisted reproductive technology outcomes in the US have persisted for black women over the last 15 years. Limited access to state mandated insurance may be contributory. Race has continued to be an independent prognostic factor for live birth and cumulative live birth rate from assisted reproductive technology in the US.

## Background

Numerous US large database registry studies have demonstrated a significant and consistent disparity in assisted reproductive technology (ART) outcomes and usage related to race/ethnicity [1–7]. An initial study of the Society for Assisted Reproductive Technology Clinic Outcome Reporting System (SARTCORS) examined racial differences between cycles from non-Hispanic black (black) and non-Hispanic white (white) women in 1999 and 2000 and noted lower pregnancy rates for black women compared to white women. Based upon the SARTCORS database, in 1999 and 2000, only 4.6% of black women underwent ART treatment while composing 12.9% of the US population while race/ethnicity was reported in only 51.6% of all SARTCORS cycles [1]. A few years later a follow up study of SARTCORS cycles from 2004 to 2006 showed a modest increase in reporting of race/ethnicity of 60% of cycles and an increase in percentage of reported cycles from black women up to 6.5%. Multivariable logistic regression analysis demonstrated that cycles from black women had almost a 33% lower chance of a live birth per-cycle-start compared to cycles from white women after controlling for confounding factors (ie. age, gravidity, etiology of infertility, number of transferred embryos) that could influence outcome [2].

Additional reports using SARTCORS [3, 7] as well as non-SARTCORS based data [8] have confirmed lower ART outcomes in black women and have reported consistent reduced odds of pregnancy for black women to be 0.62–0.63 compared to the reference group of white women. Furthermore, there was a 13.7% relative increase of live births per initiated cycle for white women in 2004–2006 compared to 1999–2000 in contrast to live birth outcomes for cycles of black women which remained essentially unchanged over the same time periods [2]. Thus, such disparities in ART outcomes had appeared to have not changed over a short period of time. A recent study from the CDC examining the National ART Surveillance System (NASS) data demonstrated that black women along with other women of color have lower than average US ART utilization rates defined as the number of ART procedures per million women of reproductive age [9]. Recognizing infertility as a disease underlies the importance of addressing such disparities and led ASRM to address and prioritize these concerns as access-to-care issues in its ongoing strategic plan [10]. Thus, the objectives of this study were to examine recent 2014–2016 data from the Society for Assisted Reproductive Technology Clinical Outcomes Reporting System (SARTCORS) database to determine if the disparities in racial ART outcomes between black and white women have changed (ie. narrowed or widened) over time and to identify possible contributing factors which may have influenced such change. In addition, as linked cycles (linking of a specific retrieval to a primary transfer either fresh or frozen/thaw) became available in 2014, we have examined the cumulative live birth rates (CLBR) per cycle-start for 2014 and 2015 as a function of race.

## Methods

### Data source and inclusion criteria

This study was exempted for review by the institutional review board of Yale School of Medicine and was approved by the SART Research Committee. A retrospective, cohort study was conducted using 2014–2016 data and compared with analyses previously performed and published using 2004–2006 [2] and 1999–2000 data [1]. De-identified data from the SARTCORS containing comprehensive data from member clinics and including more than 91% of all reported ART cycles in the United States during 2014–2016 were analyzed. Data were collected and validated by SART and reported to the Centers for Disease Control and Prevention in compliance with the Fertility Clinic Success Rate and Certification Act of 1992 (Public Law 102–493). The data in SARTCORS were validated annually with some clinics having on-site visits for chart review based on an algorithm for clinic selection. During each visit, data reported by the clinic were compared with information recorded in the patient’s charts. Ten out of 11 data fields selected for validation were found to have discrepancy rates of less than or equal to 5% [11]. Clinics submitted information about ART treatment cycles and outcomes of autologous (fresh and frozen), non-donor embryo cycles and donor oocytes according to a standardized protocol that included prompts for designing Hispanic ethnicity and indicating race as white, Asian, black, Native American or other.

To create the study data set, the SART data vendor (Redshift Technologies) selected the 563,730 ART cycles without preimplantation genetic diagnosis or screening reported by SART member clinics during the 2014–2016 study period and then excluded 1753 cycles (0.3%) to limit the study data set to clinics providing 50 or more cycles in a given year. Clinics with 50 or fewer cycles were excluded to avoid skewing data because of small sample sizes and outcomes not representative of larger SART clinics. There was missing data for race/ethnicity in 219,351 cycles (39%) which were excluded from the remaining 561,977 cycles limiting the study data set to clinics that reported race/ethnicity in all cycles. We compared the 219,351 excluded cycles because of missing data on race with the remaining 561,977 cycles and found that the live birth rates per cycle-start were essentially the same with 24.8% for both cycles with and without reported race. One hundred seventy-seven thousand five hundred four of these 561,977 remaining cycles were fresh autologous cycles. For the purpose of analysis, 11,530 fresh cycles (6.5%) were then excluded as they used donor oocytes. Also, 2530 fresh non-donor cycles (0.02%) were excluded because race/ethnicity was reported in more than one category. This left 163,444 fresh autologous, non-donor embryo cycles reported in 2014–2016 that met study inclusion criteria. In addition, we examined cumulative live birth rates linked to primary transfer for 2014 and 2015 cycles. This cumulative live birth rate analysis included 12,133 cycles which had been reported for black women and 101,672 cycles for white women. Of note, cycles in which patients identified as multi-racial were excluded to eliminate this source of confounding information. A subset analysis was performed for cycles done in mandated states which were defined as states mandating third party-payer coverage of ART during the study period of 2014–2016. These eight states included Maryland, Arkansas, Massachusetts, Rhode Island, Hawaii, Illinois, New Jersey, Connecticut.

### Statistical analysis

Data were analyzed using R 3.5.1 package for Windows (Microsoft, Redmond, WA). The treatment cycle was the unit of analysis because personal identifiers were not included and thus, precluded analysis by individual patient. Data among women with no prior ART (ie. initial cycle) were examined separately because these cycles most likely represent individual women and most likely represented cycles with a more favorable prognosis than cycles associated with a prior failed cycle. Diagnoses were examined individually to avoid obscuring relationships by using mutually exclusive categories, such as multiple female factors or multiple male and female factors. Extreme values of FSH dosage (> 80 ampules) that may have been coding errors were replaced by missing values. The implantation rate was calculated by dividing the number of fetal heartbeats on first-trimester ultrasound in a given cycle by the number of embryos transferred in that cycle. Clinical pregnancy was defined as the presence of a gestational sac by ultrasound during the first trimester. A live birth was defined as the birth of one or more living infants. Rates of both of these outcomes were calculated per cycle started**.**

A cumulative live birth was defined as the birth of one or more living infants from a linked primary transfer (fresh or frozen/thaw). SART defined its cumulative live birth outcome metric from each attempted (ie. whether actually performed or cancelled) retrieval in a given reporting year as the denominator and each live birth from any transfer, fresh or frozen, linking to that retrieval within 12 months, based on the time interval between the “Start date of the retrieval” and the “Start date of the FET”. However, due to the limitations of the de-identified dataset which did not include specific dates such as start, retrieval, and/or transfer, it was not possible to isolate the precise time interval for an FET that linked to its retrieval. Since “reporting year” (the year in which each cycle started) for each cycle was recorded, it was possible to calculate the cumulative rate within a 24-month maximum timeframe. Specifically, if a cycle with an attempt at retrieval occurred during the 2014 reporting year, any linked FET that started in 2015 was considered as a success or failure such that the two cycles (ie. the retrieval cycle and its linked FET cycle) counted as one in the numerator and one in the denominator. If an attempt at retrieval occurred during the 2015 reporting year, any linked FET that started in 2015 or 2016 that was linked to the earlier respective retrieval was considered as a success or failure. Thus, this allowed for a maximum range of 12 to 24 months depending on the year in which the linked retrieval and FET cycles occurred. If the attempt at retrieval was started in December of 2015, this would only allow a maximum of 12 months into 2016, the last year of our data, to capture an FET outcome. Attempts at retrieval and the subsequent linked FET cycles from those 2016 attempt at retrieval were excluded from our analysis for calculating cumulative live birth rate because our dataset was limited to cycles started through the end of 2016 without further follow up data into 2017.

All statistical tests were two-tailed using a *p* value of < 0.05. Percentages in specific analyses did not total to 100 because of rounding, and there were different numbers of cycles in some analyses because of missing data. Categorical values were compared using Chi-squared testing. Continuous variables were compared using t test; if the distributions were skewed, Mann-Whitney test was used. Similar to previously reported data and to facilitate comparisons, 95% confidence intervals were used. To estimate the independent contribution of race to treatment outcomes, multivariable logistic regression analyses were performed by adjusting for confounders specifically, age, parity, BMI, etiology of infertility, ovarian reserve, cycle cancellation, past spontaneous abortion, use of ICSI and number of embryos transferred.

## Results

Among the 163,444 fresh autologous, non-donor cycles, 109,004 (66.7%) cycles were among white, non-Hispanic women and 13,717 (8.4%) cycles were among black, non-Hispanic women. We excluded from further analysis 26,958 (16.5%) cycles among Asian, non-Hispanic women, 12,886 (7.9%) cycles among Hispanic women of any race, 366 (0.2%) cycles among American Indian or Alaska Native women, 513 (0.3%) cycles among Hawaiian. This resulted in analyzing 122,721 cycles using fresh autologous, non-donor embryo cycles of which 13,717 cycles (8458 initial cycles and 5259 had prior cycles) were from black women and 109,004 cycles (64,878 initial cycles and 44,126 had prior cycles) were from white women undergoing ART.

Race/ethnicity was reported with similar frequency per cycle with each successive year that was analyzed: 62.4% in 2014, 60.3% in 2015, 61.1% in 2016 with a mean of 61% over the 3 year period of 2014–2016. The reporting frequency of race/ethnicity was comparable with 60.2% which was recorded in 2004–2006 and more favorably compared with 51.6% recorded in 1999 and 2000. There was essentially no change in reporting cycle race/ethnicity in the SARTCORS database since 2006. Cycles from black women represented 8.4% of the total cycles (an increase from the 6.5% reported in 2004–2006 and a notable increase from the 4.6% reported in 1999 and 2000) while cycles from white women represented 66.7% (a decrease from the 76.8% reported in 2004–2006 and a significant decrease from 85.4% in 1999 and 2000) of total reported autologous, non-donor embryo cycles with known race/ethnicity. A woman’s race was the same as the man’s race 73.6% in 2014–2016 compared with 90.5% in 2004–2006 and 99.3% in 1999 and 2000 (*P* < 0.001). Cycles in black and white women continued to show an increase in number since reporting in 2004–2006 while the proportionate increase was greater in black than white women.

**Fig. 1:**
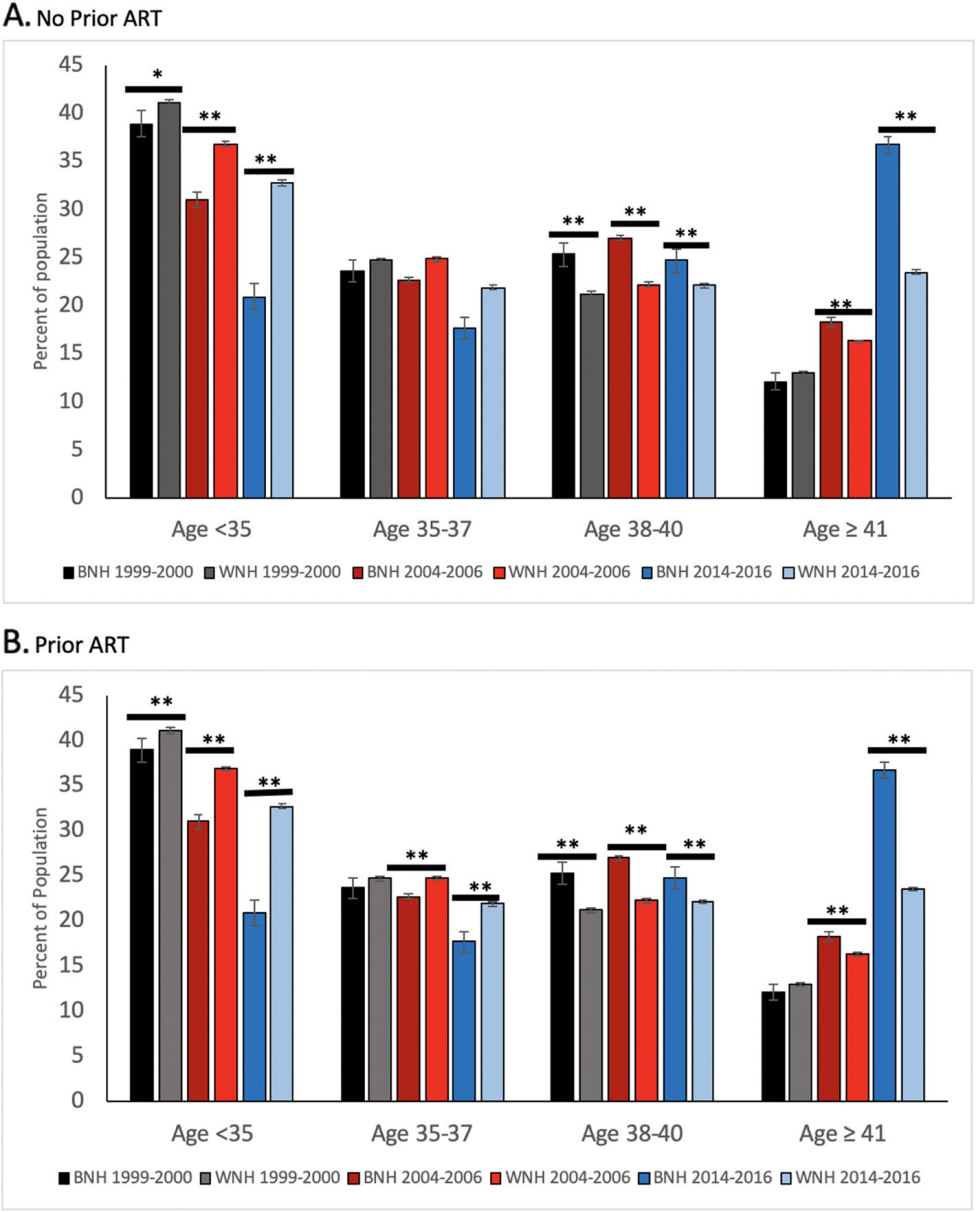
Age distribution over three time periods (1999–2000; 2004–2006; 2014–2014) for patients with no prior ART (**a**) and with prior ART (**b**). Solid black bar = BNH from 1999 to 2000, solid gray bar = WNH from 1999 to 2000, dark red bar = BNH from 2004 to 2006, light red bar = WNH from 2004 to 2006, dark blue bar = BNH from 2014 to 2016, light blue bar = WNH from 2014 to 2016. * *p* < 0.01, **p < 0.001, results reported with standard error bars and were not significant unless otherwise specified

The greatest increase in representation was primarily among older black women in the age groups of ≥ 38 with the most concentrated increase in those ≥ 41 years of age compared to white women in the same age categories (*P* < 0.001). These results are outlined in the last 2 sets of histograms (ages 38–40 and age ≥ 41) in Fig. 1a and b. During the same period of time, a smaller percentage of black women had their initial cycle before age 35 compared with white women from 2014 to 2016 (38.5% versus 54.6%, respectively; *P* < 0.001). This was also the case for black women who were less than 35 and undergoing ART with a prior cycle compared with that of white women (20.9% versus 32.7%, respectively; *P* < 0.001). This is shown in the first set of histograms (age < 35 in Fig. 1a and b).

**Fig. 2:**
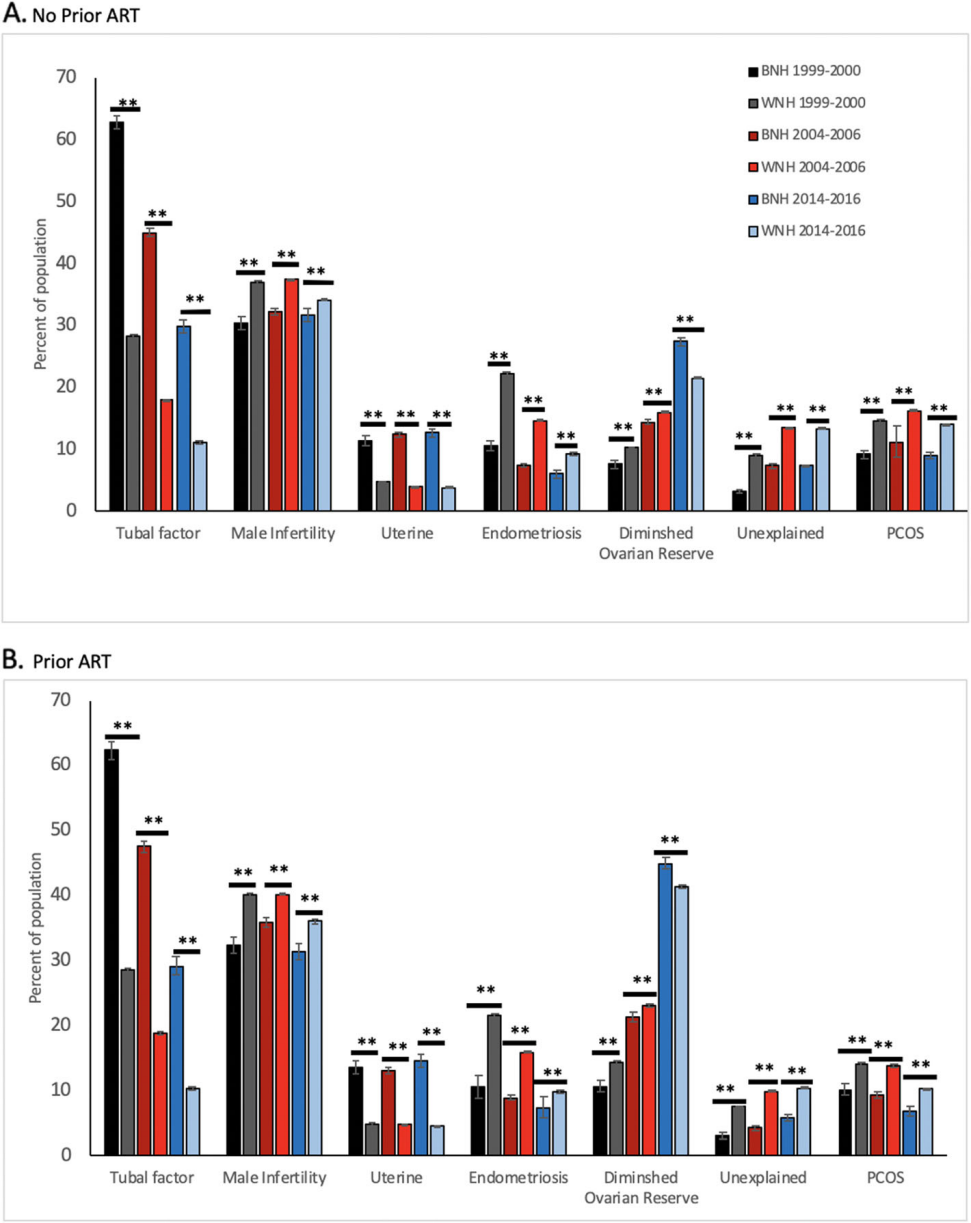
Etiologies of infertility over three time periods (1999–2000; 2004–2006; 2014–2014) for patients with no prior ART (**a**) and with prior ART (**b**). Solid black bar = BNH from 1999 to 2000, solid gray bar = WNH from 1999 to 2000, dark red bar = BNH from 2004 to 2006, light red bar = WNH from 2004 to 2006, dark blue bar = BNH from 2014 to 2016, light blue bar = WNH from 2014 to 2016. * *p* < 0.01, **p < 0.001, results reported with standard error bars and were not significant unless otherwise specified

These findings were consistent with black women having a greater proportion of a diagnosis of diminished ovarian reserve (DOR) infertility compared to white women (27.4% versus 21.5%; P < 0.001) in 2014–2016. The trend of increasing DOR infertility has continued to increase over time for both black and white women, but it has increased more dramatically among black women (Fig. 2a and b). Biomarkers of ovarian reserve were consistent with the diagnosis of diminished ovarian reserve by showing similar percentages of cycles from black compared to white women associated with low levels of Anti-Mullerian Hormone (AMH) < 1 ng/ml (in women younger than 40) and higher percentages of cycles with elevated day 3 FSH IU/L (day FSH ≥ 10) (Table 1). These observations are consistent with the increased amount of gonadotrophins used in a greater percentage of cycles from black women (Table 1).

**Table 1:** Baseline characteristics, treatment, and outcomes for fresh, nondonor cycles among black and white women

	**No Prior ART**			**Prior ART**		
	**Black (*n*** **= 8458)**	**White (*n*** **= 64,878)**		**Black (*n*** **= 5259)**	**White (*n*** **= 44,126)**	
**Characteristics (% reporting)**	**% (95% CI)**	**% (95% CI)**	***p***	**% (95% CI)**	**% (95% CI)**	***p***
Women’s age (year)			< 0.001			< 0.001
< 35	38.5 (37.5–39.6)	54.6 (54.2–55)		20.9 (19.8–22.1)	32.2 (32.2–33.1)	
35–37	22.4 (21.5–23.3)	21.2 (20.8–21.5)		17.7 (16.6–18.7)	21.9 (21.5–22.3)	
38–40	19.8 (18.9–20.6)	15.2 (14.9–15.4)		24.7 (23.6–25.9)	22.1 (21.7–22.4)	
41–42	10.1 (9.4–10.7)	5.7 (5.5–5.8)		16.3 (15.3–17.3)	11.8 (11.5–12.1)	
> 42	9.3 (8.7–9.9)	3.5 (3.3–3.6)		20.4 (19.3–21.5)	11.7 (11.4–12)	
BMI > =30	47.4 (46.4–48.5)	31.5 (31.1–31.9)	<.001	49.4 (48–50.7)	34.5 (34.1–35)	< 0.001
Nulliparous	55.6 (54.1–57.1)	51.2 (50.5–51.8)	< 0.001	53.3 (51.7–55)	44.4 (43.8–44.9)	< 0.001
Past spontaneous abortions	28.6 (27.6–29.6)	20.2 (19.9–20.5)	< 0.001	41.1 (39.8–42.5)	32.8 (32.4–33.3)	< 0.001
Diagnosis						
Tubal Factor	29.8 (28.8–30.8)	11 (10.8–11.2)	< 0.001	29.1 (27.8–30.3)	10.4 (10.2–10.7)	< 0.001
Tubal Ligation	3.2 (2.8–3.6)	1.7 (1.6–1.8)	< 0.001	2.1 (1.7–2.5)	1 (0.9–1.1)	< 0.001
Hydrosalpinx	2.9 (2.6–3.3)	0.8 (0.7–0.8)	< 0.001	2.9 (2.5–3.4)	0.7 (0.6–0.8)	< 0.001
Other	24.2 (23.3–25.2)	8.6 (8.4–8.9)	< 0.001	24.5 (23.4–25.7)	8.8 (8.6–9.2)	< 0.001
Male Infertility	31.6 (30.6–32.6)	34.2 (33.9–34.6)	< 0.001	31.3 (30.1–32.6)	36 (35.5–36.4)	< 0.001
Uterine Factor	12.6 (11.9–13.4)	3.8 (3.6–3.9)	< 0.001	14.5 (13.6–15.5)	4.5 (4.3–4.7)	< 0.001
History of endometriosis	6 (5.5–6.5)	9.2 (9–9.4)	< 0.001	7.4 (6.7–8.1)	9.8 (9.5–10.1)	< 0.001
Diminished ovarian reserve	27.4 (26.4–28.3)	21.5 (21.2–21.8)	< 0.001	44.9 (43.5–46.2)	41.3 (40.8–41.7)	0.73
Unexplained	7.3 (6.7–7.9)	13.3 (13–13.6)	< 0.001	5.7 (5.1–6.4)	10.4 (10.1–10.7)	< 0.001
Ovulation Disorder (PCOS)	9 (8.4–9.6)	13.9 (13.7–14.2)	< 0.001	6.8 (6.1–7.5)	10.2 (10–10.5)	< 0.001
Other	22.5 (21.6–23.4)	25.5 (25.1–25.8)	< 0.001	20.4 (19.3–21.5)	21.2 (20.9–21.6)	0.15
Metrics of ovarian reserve						
Day 3 FSH > =10 (IU/L)	71.8 (70.8–72.8)	65.4 (65.1–65.8)	< 0.001	81.2 (80.1–82.2)	74 (73.6–74.4)	< 0.001
Mean Day 3 FSH (IU/L)	11.5 (11.2–11.83)	11.7 (11.65–11.75)	0.217	11.6 (11.55–11.65)	12.0 (11.7–12.2)	0.024
FSH dosage > = 37 ampules (91.7)	59 (57.9–60.1)	52.2 (51.8–52.6)	< 0.001	69 (67.7–70.4)	64.8 (64.3–65.2)	< 0.001
Mean AMH (ng/mL)	3.2 (3.1–3.3)	3.4 (3.4–3.4)	0.003	2.1 (2.0–2.2)	2.3(2.3–2.3)	< 0.001
AMH < 1 among women< 40 yr	13.4 (12.6–14.3)	13.4 (13.1–13.6)	0.95	21.7 (20.2–23.3)	21 (20.6–21.5)	0.4
High ovarian response	0.8 (0.7–10.6)	0.5 (0.4–0.5)	< 0.001	1 (0.8–1.3)	0.7 (0.7–0.8)	0.049
Mean oocytes retrieved	13.8 (13.6–14.0)	14.3 (14.2–14.4)	< 0.001	14.4 (14.1–14.7)	14.5 (14.4–14.6)	0.52
Mean embryos cryopreserved	2.70 (2.61–2.79)	2.75 (2.72–2.78)	0.24	1.29 (1.22–1.36)	1.30 (1.28–1.32)	0.77
Cycle Cancelled	12.9 (12.2–13.6)	9.9 (9.6–10.1)	< 0.001	17.1 (16.1–18.1)	13.3 (13.0–13.6)	< 0.001
Due to low response	76.4 (73.8–78.9)	84.1 (83.2–85)	< 0.001	77 (74.1–79.7)	80.1 (79.1–81.1)	0.03
ICSI	57.7 (56.7–58.8)	61.2 (60.8–61.6)	< 0.001	58.3 (56.9–59.6)	63.9 (63.4–64.3)	< 0.001
No of embryos			< 0.001			0.018
1	34.9 (33.6–36.3)	38.2 (37.7–38.6)		25.6 (24–27.3)	26.3 (25.8–26.8)	
2	53.4 (52–54.8)	54.1 (53.6–54.6)		51.5 (49.7–53.4)	53.1 (52.5–53.7)	
3+	11.7 (10.8–12.6)	7.7 (7.5–8)		22.8 (21.3–24.4)	20.6 (20.1–21.1)	
No. of embryos transferred: mean (sd)	1.8 (0.74)	1.7 (0.67)	< 0.001	2.06 (0.9)	2.02 (0.88)	0.03
Implantation rate %: mean (sd)	72.4 (32.4)	76.5 (33.4)	< 0.001	63.9 (35.3)	67.1 (34.1)	0.01
Treatment outcome						
Clinical intrauterine gestation (CIG)	24.4 (23.5–25.3)	33 (32.7–33.4)	<.001	17.2 (16.2–18.2)	25.1 (24.7–25.3)	< 0.001
Spontaneous abortion	21 (19.3–22.8)	14.5 (14–15)	0.2	27.5 (24.6–30.5)	18.6 (17.8–19.3)	<.001
Live birth per CIG	76.4 (74.5–78.2)	84.6 (84.1–85)	<.001	69.8 (66.6–72.7)	80.5 (79.7–81.2)	< 0.001
Biochemical pregnancy	4.8 (4.4–5.3)	5.7 (5.5–5.9)	0.0013	4.2 (3.7–4.8)	5.7 (5.5–6)	< 0.001
Ectopic or heterotopic	0.5 (0.4–0.7)	0.5 (0.5–0.6)	0.75	0.3 (0.1–0.5)	0.5 (0.4–0.6)	0.02
Not pregnant	58.3 (57.2–59.4)	47.2 (46.8–47.6)	<.001	68.4 (67.1–70)	58.3 (57.8–58.7)	< 0.001
Live birth per cycle started	18.6 (17.8–19.5)	27.9 (27.6–28.3)	<.001	12 (11.1–12.9)	20.2 (19.8–20.6)	< 0.001
Plurality of birth (24)			<.001			< 0.001
Singleton	76 (73.8–78)	76.9 (76.2–77.5)		75.2 (71.7–78.5)	75.8 (74.9–76.6)	
Twins	21.2 (19.2–23.3)	21.9 (21.3–22.5)		21.1 (18–24.5)	22.6 (21.8–23.5)	
Triples or more	2.8 (2.1–3.8)	1.3 (1.1–1.5)		3.7 (2.4–5.5)	1.6 (1.3–1.9)	
eSET (%) < 38 y/o (44.1)	21 (19.9–22.2)	23.9 (31.5–31.9)	<.001	12.7 (11.3–14.2)	13.9 (13.4–14.3)	0.16
States						
Mandated	34.7 (33.7–35.7)	27.7 (27.4–28.1)	<.001	39.8 (38.4–41.1)	32.4 (32–32.9)	<.001
Non-mandated	65.1 (64.1–66.1)	71.5 (71.1–71.8)	<.001	60.2 (58.9–61.5)	67 (66.5–67.4)	<.001

The mean BMI for cycles in black women was greater than from white women. Both mean BMIs are within the range of “overweight” (25.0–29.9), but at more extreme ends of the spectrum of that range. In addition, the percentage of black versus white initial ART cycles that were associated with a BMI ≥ 30 kg/m2 (obese) was 47.4% versus 31.5%, respectively higher among black women (*P* < 0.001). This was similar for black women with a previous ART cycle.

When examining the etiology of infertility, women demonstrated significant differences between race (Fig. 2a and b). The proportion of reproductive organ pathology such as tubal or uterine pathology was greater among cycles from black women. Although the percentage of infertility due to tubal factor in women undergoing ART had decreased over time for women of both races since 1999 and 2000, it remained high among black women. Among women with no prior ART, black women in 2014–2016 were 2.7 times more likely than white women to have tubal factor (29.8% versus 11% respectively; *P* < 0.001). Thus, tubal factor continued to have an increasing widening gap as an etiology of infertility over time for women with no prior ART. Similar trends were noted between races for women who had prior ART (Fig. 2b). Initial cycles and cycles preceded by prior ART cycles from black women continued to have greater than 3 times the occurrence of uterine factor infertility compared to white women (*P* < 0.001) as has been the trend since 1999 and 2000.

Specific etiologies of infertility (unexplained infertility, male factor, endometriosis and PCOS) showed greater representation among cycles from white compared to cycles from black women as illustrated in Fig. 2a and b. Interestingly, the diagnosis of unexplained infertility seemed to have plateaued in both black and white women over the last 10 years, and was a diagnosis 1.8 times more common among white women (*P* < 0.001). Differences in the diagnosis of unexplained infertility remained significantly greater in 2014–2016 among cycles from white than black women for both initial cycles and cycles preceded by prior ART cycles (*P* < 0.001).

The occurrence of male factor, endometriosis and PCOS in 2014–2016 were slightly greater among white than black women (34.2% compared to 31.6%, respectively for male factor (*P* < 0.001); 9.2% compared to 6%, respectively for endometriosis (*P* < 0.001) and 13.9% compared to 9%, respectively for PCOS (*P* < 0.001) (Table 1). The trend of male factor, endometriosis and PCOS having greater differences in representation among initial ART cycles from white compared to black women narrowed in 2014–2016 as compared to 2004–2006. This trend was also noted for cycles from women with a prior history of ART (Table 1).

Cancellation rates were higher among cycles from black women compared to white women in 2014–2016. A cancellation rate of initial cycles for cycles from black women was 12.9% compared with 9.9% from white women (*P* < 0.001) (Table 1). Similar cancellation rates were noted for cycles from black women with prior ART. These cancellation rates were associated with a greater percentage of cycles among black women being associated with cycle day 3 FSH ≥ 10 IU/l) compared with cycles from white women and similarly from women with a prior history of ART (Table 1). A greater percentage of cycles of black than white women were associated with a diagnosis of DOR.

Elective single embryo transfer (eSET) in women less than 38 years old occurred less frequently in cycles from black women regardless of initial or previous history of having prior ART cycle (*P* < 0.001). The overall rates of eSET in 2014–2016 were considerably higher regardless of race than previously reported in 2004–06 (*P* < 0.001) (Table 1).

**Fig. 3:**
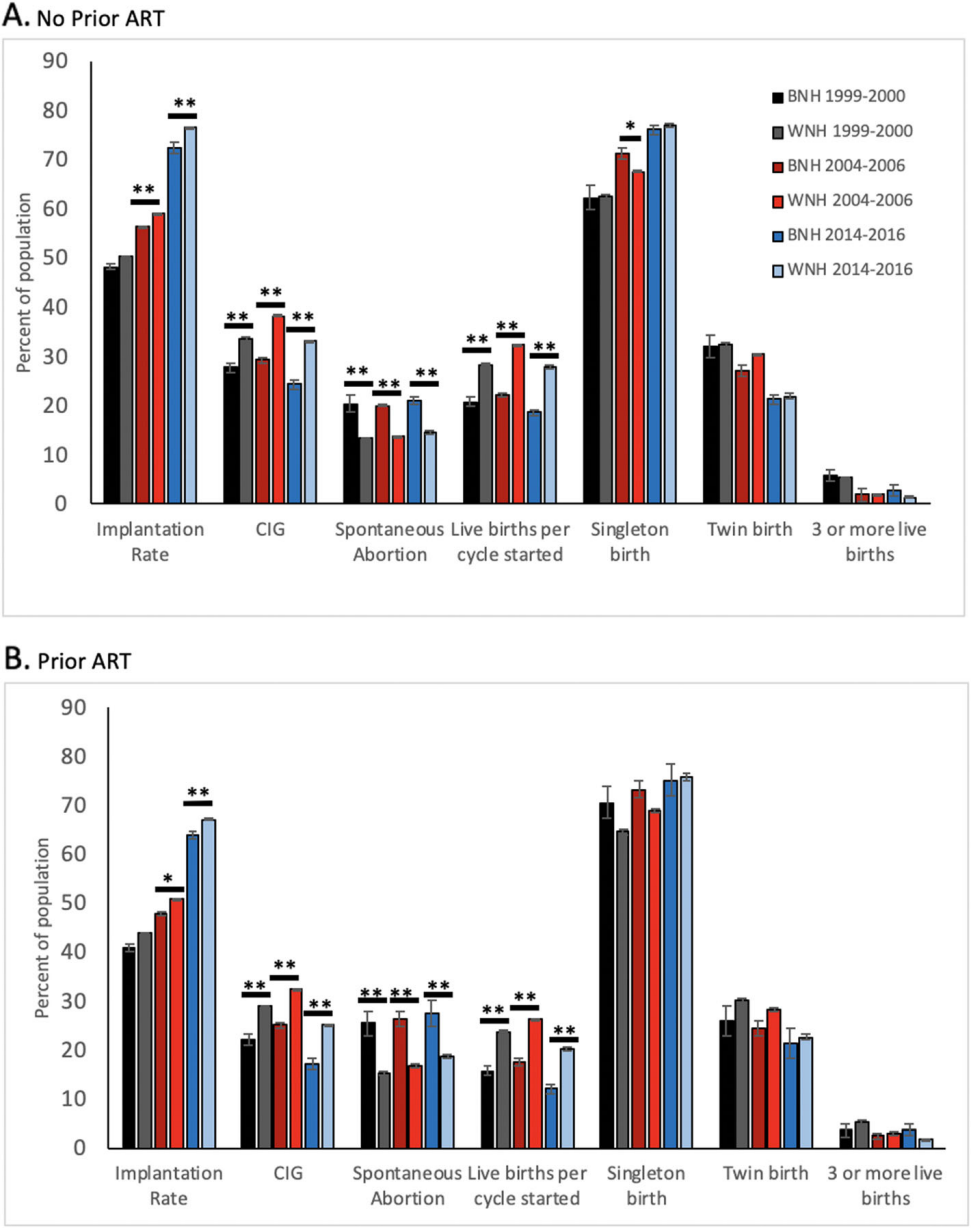
Cycle outcomes over three time periods (1999–2000; 2004–2006; 2014–2014) for patients with no prior ART (**a**) and with prior ART (**b**). Solid black bar = BNH from 1999 to 2000, solid gray bar = WNH from 1999 to 2000, dark red bar = BNH from 2004 to 2006, light red bar = WNH from 2004 to 2006, dark blue bar = BNH from 2014 to 2016, light blue bar = WNH from 2014 to 2016. * *p* < 0.01, **p < 0.001, results reported with standard error bars and were not significant unless otherwise specified

Treatment outcomes among cycles using fresh, nondonor embryos continued to be less for black women in 2014–2016 as they had been in 2004–2006 as illustrated in Fig. 3a and b. A decrease in clinical intra-uterine gestation (CIG) among black women occurred (*P* < 0.001) despite having a slightly greater mean number of embryos transferred than white women during an initial ART cycle (*P* < 0.001) or with a prior history of ART cycles (*P* = 0.03).

These findings were also accompanied by decreased implantation rates for 2014–2016 cycles from black women than white women both for initial ART cycles and with prior ART cycles as illustrated in Fig. 3a and b. Overall implantation rates in 2014–2016 were higher regardless of race than previously reported in 2004–2006 as noted in Fig. 3a and b.

Live births per cycle-start (LBR) continued to be less frequent among initial cycles from black 18.6% versus white 27.9% women (*P* < 0.001) and 12% versus 20.2% live birth per cycle started (*P* < 0.001) respectively for black and white women with prior ART cycles. Thus, the gap in LBR showed a small incremental decrease for both black and white women between 2004 and 2006 and 2014–2016 as noted in Fig. 3a and b.

Multivariable logistic analysis demonstrated that race was an independent predictor of live birth independent of age, parity, BMI, etiology of infertility, ovarian reserve (Day 3 FSH, AMH), cycle cancellation, past spontaneous abortions, use of ICSI or number of embryos transferred (Table 2). Initial cycles with fresh transfer in black women were less likely to result in a live birth than fresh transfer in initial cycles in white women (OR 0.71; *P* < 0.001) (Table 2) independent of age, parity, BMI, etiology of infertility, ovarian reserve, past spontaneous abortions, cycle cancellation, use of ICSI or number of embryos transferred. This was also the case for cycles from women with prior ART fresh transfers from black women which were less likely to result in a live birth than fresh transfer from white women (OR 0.69; *P* < 0.001) (Table 2).

**Table 2:** Independent predictors of achieving live birth and cumulative live birth

	Live birth rate for fresh nondonor cycles				Cumulative live birth rate for primary transfer (fresh or thawed FET) in cycles 2014/ 2015			
	Without prior ART		With prior ART		Without prior ART		With prior ART	
	Odds ratio (95% CI)	***p***	Odds ratio (95% CI)	***p***	Odds ratio (95% CI)	***p***	Odds ratio (95% CI)	***p***
**Race**								
**White**	Reference		Reference		Reference		Reference	
**Black**	0.71 (0.66, 0.77)	< 0.001	0.69 (0.62, 0.77)	< 0.001	0.65 (0.60, 0.70)	< 0.001	0.67 (0.61, 0.74)	< 0.001
**Women’s age (y)**								
**< 35**	Reference		Reference		Reference		Reference	
**35–37**	0.72 (0.68, 0.76)	< 0.001	0.78 (0.72, 0.83)	< 0.001	0.72 (0.68, 0.76)	< 0.001	0.79 (0.74, 0.84)	< 0.001
**38–40**	0.47 (0.44, 0.50)	< 0.001	0.55 (0.47, 0.55)	< 0.001	0.50 (0.46, 0.53)	< 0.001	0.55 (0.51, 0.59)	< 0.001
**41–42**	0.22 (0.19, 0.25)	< 0.001	0.27 (0.24, 0.31)	< 0.001	0.25 (0.22, 0.28)	< 0.001	0.35 (0.31, 0.39)	< 0.001
**> 42**	0.09 (0.07, 0.11)	< 0.001	0.10 (0.08, 0.12)	< 0.001	0.09 (0.07, 0.11)	< 0.001	0.16 (0.14, 0.19)	< 0.001
**Nulliparous**	0.89 (0.84, 0.96)	0.001	0.78 (0.73, 0.83)	< 0.001	0.92 (0.86, 1.00)	0.04	0.90 (0.84, 0.96)	< 0.001
**Past spontaneous abortions**	0.96 (0.90, 1.03)	0.2	1.02 (0.95, 1.09)	0.68	0.97 (0.91, 1.05)	0.47	1.01 (0.95, 1.07)	0.87
**Tubal factor**	1.00 (0.89, 1.14)	0.94	0.95 (0.83, 1.08)	0.44	0.92 (0.81, 1.05)	0.23	0.92 (0.82, 1.04)	0.21
**Male factor**	1.05 (1.0, 1.09)	0.05	1.06 (1.00, 1.12)	0.06	0.95 (0.90, 1.00)	0.04	0.99 (0.94, 1.05)	0.66
**Uterine factor**	0.83 (0.75, 0.92)	< 0.001	0.97 (0.85, 1.11)	0.69	0.98 (0.89, 1.09)	0.77	0.98 (0.88, 1.10)	0.53
**Diminished ovarian reserve**	0.84 (0.79, 0.90)	< 0.001	0.82 (0.76, 0.88)	< 0.001	0.74 (0.69, 0.79)	< 0.001	0.72 (0.67, 0.77)	< 0.001
**Day 3 FSH**								
**< 10 (IU/L)**	Reference		Reference		Reference		Reference	
**> =10 (IU/L)**	0.91 (0.82, 1.01)	0.07	0.91 (0.80, 1.01)	0.3	0.89 (0.79, 1.00)	0.05	0.90 (0.78, 1.03)	< 0.001
**AMH (ng/mL)**	1.36 (1.26, 1.46)	< 0.001	1.23 (1.14, 1.34)	< 0.001	2.06 (1.91, 2.24)	< 0.001	1.78 (1.64, 1.93)	< 0.001
**ICSI**	0.97 (0.92, 1.03)	0.27	0.94 (0.87, 1.02)	0.12	1.15 (1.09, 1.22)	< 0.001	1.48 (1.40, 1.57)	< 0.001
**Cycle Cancelled**	36.83 (16.2, 106)	< 0.001	40 (8.3, 7181.12)	< 0.001	1.32 (0.90, 1.89)	0.14	0.29 (0.16, 0.50)	< 0.001
**No of embryos**								
**1**	Reference		Reference		Reference		Reference	
**2**	1.28 (1.22, 1.33)	< 0.001	1.39 (1.31, 1.49)	< 0.001	0.74 (0.71, 0.78)	< 0.001	0.88 (0.83, 0.93)	< 0.001
**3+**	1.15 (1.05, 1.27)	0.004	1.16 (1.06, 1.27)	0.001	0.45 (0.41, 0.50)	< 0.001	0.50 (0.46, 0.54)	< 0.001
**BMI > =30**	0.77 (0.73, 0.81)	< 0.001	0.87 (0.81, 0.93)	< 0.001	0.71 (0.68, 0.75)	< 0.001	0.86 (0.81, 0.91)	< 0.001

We examined 2014 and 2015 linked cycles with primary transfers either fresh or frozen/thaw and noted disparities in outcomes across all SARTCORS designated age categories for cumulative live birth rates (CLBR) per cycle. Significant lower CLBR for each age category were noted for cycles from black versus white women. CLBR for initial ART cycles from black and white women age < 35 were 42.4% versus 55.0% (*P* < 0.001), age 35–37 were 32.9% versus 39.6% (*P* < 0.001), age 38–40 were 19.9% versus 26.5% (*P* < 0.001), age 41–42 were 8.1% versus 13.5% (*P* < 0.001) and age > 42 2.4% versus 4.5% (*P* = 0.02), respectively. CLBR for those with prior ART cycles from black and white women age < 35 were 31.0% versus 40.2% (*P* < 0.001), age 35–37 were 25.5% versus 31.4% (*P* < 0.001), age 38–40 were 14.9% versus 20.7% (*P* < 0.001), age 41–42 were 8.0% versus 11.9% (*P* < 0.001) and age > 42, 2.9% versus 4.3% (*P* = 0.02), respectively. CLBR per cycle were less regardless of race across age groups for those with prior cycles compared to those with no prior cycles reflecting inherent differences in prognosis for expected success between groups.

Multivariable logistic analysis demonstrated that race was an independent predictor of cumulative live birth after controlling for age, parity, BMI, etiology of infertility, ovarian reserve (Day 3 FSH, AMH), cycle cancellation, past spontaneous abortions, use of ICSI or number of embryos transferred (Table 2). Initial cycles with primary transfer (whether fresh or frozen/thaw transfer) in black women were less likely to result in a live birth than primary transfer in initial cycles of white women (OR 0.64; *P* < 0.001) (Table 2). This was independent of age, parity, BMI, etiology of infertility, ovarian reserve, cycle cancellation, past spontaneous abortions, use of ICSI or number of embryos transferred. This was also the case for cycles from women with prior ART with primary transfers from black women which were less likely to result in a cumulative live birth than primary transfer in white women (OR 0.67; *P* < 0.001) (Table 2).

Furthermore, we also examined the question of whether there were any racial differences in proportional representation (possible disparity in access) and/or LBR/CLBR (disparity in outcome) if the ART cycle had been performed in a mandated versus non-mandated state (Table 1). In mandated states 13.5% of cycles were from black women and 86.5% of cycles were from white women. This was consistent with the demographic representation of the population of black (14%) and white (86%) women of reproductive age (ie. 18–44 years old in 2016 living in mandated states). However, in non-mandated 10.2% of cycles were from black women and 89.7% of cycles were from white women. This was in contrast less proportionate to the demographic representation of black (20%) and white (80%) women of reproductive age living in non-mandated states [12]. Thus, there was considerably less representation of cycles from black women compared to white women relative to their demographic representation in non-mandated compared to mandated states (*P* < 0.001). Although similar LBR were noted in cycles from black and white women regardless of whether or not the cycle was performed in a mandated or non-mandated state, this was not the case for CLBR. Cycles from black women had a higher CLBR in mandated compared to non-mandated states 24.9% versus 22.7%, respectively (*P* = 0.006) despite cycles from white women demonstrating no difference in CLBR regardless of state insurance status (35.1% versus 35.7%, respectively).

## Discussion

This is the first study using the SARTCORS national database that has examined long-term trends in racial disparities between black and white women with respect to ART treatment cycle outcomes using autologous non-donor embryos. Additionally, this is also the first study that has examined cumulative live birth rates (CLBR) per cycle in the context of racial disparities as linked cycles (retrieval linked with primary transfer whether fresh and/or frozen/thaw transfer) first became available in the SARTCORS database in 2014. Race has continued to be an independent prognostic factor for live birth rate from ART.

These data demonstrate a persistent racial gap with respect to ART outcomes compared to 1999–2000 and 2004–2006 and may suggest issues of access based in part upon the lack of uniform availability of state insurance mandates for ART. Specifically, increased age and/or BMI at time of treatment, greater prevalence of pelvic pathology known to be associated with lower implantation rates and less proportionate representation to the population as a whole among those undergoing ART care in non-mandated insurance states may contribute to outcome disparities between black and white women which have persisted over the past decade. Interestingly, there has been no substantial change in reporting frequency of race/ethnicity over the most recent ten-year period. This was unexpected given that race/ethnicity has been a mandatory field in the SARTCORS database.

Since 1999–2000 there has been a continued increase in the percentage of reported autologous, non-donor embryo cycles from women regardless of race while there had been an incremental increase in the percentage of cycles from black women. The frequency of a woman’s race matching the same race as her partner has also decreased with time. These specific changes most likely reflect the general demographic trend in the US over the last two decades towards a more heterogenous society [13]. Although the percentage of cycles from black women have increased, the greatest increase has been in older women as illustrated by a concomitant decrease in cycles from black women < 35 years old and an increase in cycles from women ≥ 38 years old as illustrated in Fig. 1a and b. Such changes in representation at the demographic extremes of the age spectrum exacerbated and continued the widening of the age gap between black and white women since 1999 and 2000 resulting in a greater proportion of black women generally older than white women who underwent ART. This increase in cycles from black women among an older demographic since 1999–2000 may represent several possible reasons which include a rise in socioeconomic status, an increase in general awareness of advancements in reproductive technologies, an increase in access through some states that have insurance mandates, less reluctance on part of themselves or their partner to pursue advanced technologies to conceive and perhaps a more general acceptance of egg donation among white women of advanced reproductive age.

Thus, the overall long term trend shows a rise in an increasing age gap between cycles from black and white women undergoing ART. As expected, this increasing age gap between races was accompanied by a widening racial gap in the diagnosis of diminished ovarian reserve. In addition, these age disparities were accompanied by an elevated mean BMI in black compared to white women which has been demonstrated to be associated with significant disparities in live birth rates between those with normal and elevated BMI [5]. This was also accompanied by greater cancellation rates from black women. This finding of lower ovarian reserve was consistent with previous studies showing differences in serum AMH noted between black and white women [14, 15].

Reproductive organ pathology such as tubal or uterine pathology continued to be significantly higher among cycles from black than white women. However, tubal factor represented a decreasing indication for ART reflecting a change in the patient population and etiology for infertility over time of women who undergo ART. This may also suggest that tubal and uterine pathology may be amenable to surgical correction prior to cycle of ART (ie. myomectomy for fibroids impacting the endometrial cavity or salpingectomy for hydrosalpinx) in order to potentially narrow the disparity gap in ART outcomes.

The overall rates of eSET in 2014–2016 were considerably higher regardless of race than previously reported in 2004–2006 although eSET occurred less frequently in cycles from black women compared to white women regardless of initial or previous history of having prior ART cycle. An increase in eSET reflects greater effort in recent years to intentionally move toward greater frequency of eSET in a successful effort to reduce the multiple birth rates. As black women were older they were less likely to have met criteria for having an eSET [16].

It is speculated that decreased quality of embryos in cycles from older black women may be attributed to embryos created from older oocytes and thus resulting in greater aneuploidy embryos. While issues concerning decreased quality of endometrium may be speculated to be attributed to increased pelvic pathology (ie. fibroids and hydrosalpinx) noted in cycles in black women with greater frequency thus, contributing to less receptive endometrium and reduced implantation. As fresh ART cycles from black women in 2014–2016 tended to include a greater proportion of older women with accompanied diminished ovarian reserve and higher BMI compared to women with cycles in 2004–2006, the gap in live birth per cycle start minimally narrowed between black and white women in 2014–2016 compared to 2004–2006.

Two thousand fourteen and 2015 linked cycles with primary transfers either fresh or frozen/thaw of retrieval demonstrated significant disparities in outcomes across all age groups for cumulative live birth (CLBR) rates per cycle. Significant lower CLBR for each age category were noted for cycles from black versus white women. CLBR from initial ART cycles and from those with prior ART cycles in black women were 64 and 67%, respectively as likely as white women to result in a live birth following primary transfer. The outcomes as expressed by CLBR show greater disparity in outcome with advancing age. CLBR per cycle were generally less regardless of race across age groups for those with prior cycles compared to those with no prior cycles again reflecting inherent differences in prognosis for expected success between those having initial cycles and those with prior unsuccessful cycles.

There appears to have been a greater racial disparity of proportionate representation of cycles whether they took place in mandated states or non-mandated states and corroborates previous observations of others examining the issue of ART access using non-SARTCORS databases [9]. Given that there is no difference in self-reported infertility rates by race/ethnicity [17] the fact that there is a difference in participation is a compelling observation underlying a modifiable disparity in potential access. This is furthermore emphasized by the self-reported differences in fewer visits for infertility care noted by underinsured women [17]. It has been stated that insurance coverage of ART is associated with higher utilization and improvements in practice [9, 18, 19]. However, this also raises the question of whether this disparity in participation represented a difference in economic access to ART [20–22] and/or whether other unidentified social and/or culture factors associated with race underlined such a difference [23, 24]. Despite these observations of disparity in access, cycle LBRs (outcomes) were not significantly different between black and white women based upon the presence or absence of state insurance mandates. However, CLBR were higher in mandated compared to non-mandated states for cycles from black women. This further begs the question of whether expansion of state mandates for ART would narrow the racial disparity gap for access and outcome for this highly specialized care.

Recognition of the strengths and limitations of using SARTCORS datasets to examine racial disparities in ART outcomes is important to acknowledge. Utilizing a standardized, large de-identified, validated national database allows for the statistical power of using large sample sizes to identify changes and trends over years that may go unrecognized in smaller datasets. However, as comprehensive as this dataset is it is confounded by missing data. Specifically, 39% of reported cycles were missing data regarding identifying race despite it being a mandatory field. In addition, this database does not record socioeconomic status (ie. annual salaries) or proxies for it (ie. highest obtained educational level) which remains an important confounding variable when studying the impact of race. Additionally, race itself is self-reported in the SARTCORS dataset and becomes more subjective as we as a society become more diverse and heterogeneous over time. Recognizing these strengths and limitations of the SARTCORS database are useful when future efforts are made when testing hypotheses such as does race determine live birth rate or are socioeconomic conditions that inherently limit access to health services such as ART lead to poorer reproductive outcomes***.***

These data indicate that race remains a persistent independent stratifying factor of outcome and access to ART in the US. These data further suggest that several factors may have contributed to these disparities. We believe that the persistent disparity gap in ART live birth rates as well as cumulative live birth rate (outcomes) between black and white women are unlikely to meaningfully decrease without proactive effort. Such strategies for future consideration might include addressing issues of education of segments of the population about greater age (age-related infertility) and BMI as detrimental influences to outcome, a thoughtful therapeutic approach to specific concomitant pelvic pathology and consideration of expanded availability of insurance coverage. Greater effort should be made to report race in SARTCORS to insure capturing the most complete data which may allow for the development of more informative approaches to these important issues. Such insights could lead to strategic approaches that potentially narrow the racial disparity gap in ART treatment outcomes and be further evaluated for their relative strength of effectiveness.

## Data Availability

The data that support the findings of this study are available from SARTCORS but restrictions apply to the availability of these data, which were used under license for the current study, and so are not publicly available. Data are however available from the authors upon reasonable request and with permission of SARTCORS.
