# Correction to: Status of racial disparities between black and white women undergoing assisted reproductive technology in the US

**DOI:** 10.1186/s12958-020-00683-z

**Published:** 2020-12-21

**Authors:** David B. Seifer, Burcin Simsek, Ethan Wantman, Alexander M. Kotlyar

**Affiliations:** 1 Department of Obstetrics, Gynecology and Reproductive Sciences, Yale School of Medicine, 330 Cedar St, New Haven, CT 06510 USA; 2 Department of Statistics, University of Pittsburgh, Pittsburgh, PA 15260 USA; 3 Redshift Technologies, New York, NY 10016 USA

**Correction to: Reprod Biol Endocrinol (2020) 18:113**

**https://doi.org/10.1186/s12958-020-00662-4**

Following publication of the original article [1], the authors reported an error in the author names. In the current presentation the given names are presented in initials. The authors want to correct the error and present the authors given names in full. See below correct presentation.

David B. Seifer, Burcin Simsek, Ethan Wantman and Alexander M. Kotlyar

The original article has been corrected.
