# Correction to: Status of racial disparities between black and white women undergoing assisted reproductive technology in the US

**DOI:** 10.1186/s12958-021-00806-0

**Published:** 2021-07-30

**Authors:** David B. Seifer, Burcin Simsek, Ethan Wantman, Alexander M. Kotlyar

**Affiliations:** 1 Department of Obstetrics, Gynecology and Reproductive Sciences, Yale School of Medicine, 330 Cedar St, New Haven, CT 06510 USA; 2 Department of Statistics, University of Pittsburgh, Pittsburgh, PA 15260 USA; 3 Redshift Technologies, New York, NY 10016 USA

**Correction to: Reprod Biol Endocrinol 18, 113 (2020)**

**https://doi.org/10.1186/s12958-020-00662-4**

Following publication of the original article [1], the keywords term “racial disparities ART, racial disparities IVF, IVF racial disparities, and ART racial disparities” were not included. The keywords have been added.

The original article [1] has been updated.
